# Application of Nanodiamonds in Biomolecular Mass Spectrometry

**DOI:** 10.3390/ma3031845

**Published:** 2010-03-15

**Authors:** Xianglei Kong, Ping Cheng

**Affiliations:** 1 Department of Chemistry and Chemical Biology, Cornell University, Ithaca, NY 14850, USA; 2 The State Key Laboratory of Elemento-organic Chemistry, Nankai University, Tianjin 300071, China; 3 The School of Environmental and Chemical Engineering, Shanghai University, Shanghai 200444, China; E-Mail: Pingcheng@shu.edu.cn

**Keywords:** nanodiamonds, mass spectrometry, matrix assisted laser desorption/ionization, solid phase extraction, peptide, protein, DNA oligonucleotide, body fluid, non-covalent interaction, organic functionalization

## Abstract

The combination of nanodiamond (ND) with biomolecular mass spectrometry (MS) makes rapid, sensitive detection of biopolymers from complex biosamples feasible. Due to its chemical inertness, optical transparency and biocompatibility, the advantage of NDs in MS study is unique. Furthermore, functionalization on the surfaces of NDs expands their application in the fields of proteomics and genomics for specific requirements greatly. This review presents methods of MS analysis based on solid phase extraction and elution on NDs and different application examples including peptide, protein, DNA, glycan and others. Owing to the quick development of nanotechnology, surface chemistry, new MS methods and the intense interest in proteomics and genomics, a huge increase of their applications in biomolecular MS analysis in the near future can be predicted.

## 1. Introduction

As a product of the detonation of carbon-based explosives, nanodiamond (ND) was firstly formed in the 1960s by Russian researchers [1]. However, for some reasons, it remained concealed for many years. It was not until the late 1980s that researchers found some interesting aspects of this material [2]. Since then, ND interest has rapidly increased, and expanded into many different fields [3,4,5,6]. Due to its chemical inertness, hardness, optical transparency and biocompatibility, ND has been studied and applied in a variety of research fields, such as analytical chemistry, biology, catalysis, spectroscopy, materials, electronic applications and quantum computing [3]. For example, NDs can emit single photon luminescence when stimulated by laser, owing to different point defects including nitrogen-vacancy, silicaon-vacancy and nickel-nirogen complexes. Due to its low cytotoxicity and no sign of photobleaching, this material has become a very important fluorescent probe for intracellular processes and has promise in drug delivery for cancer therapeutics [7,8,9,10]. As such a rediscovered material, ND and its applications have recently been prospected and reviewed by several researchers [3,4,5,6]. Individually, in this review, we will just focus on its application in the field of biomolecular mass spectroscopic analysis.

As one of the most routinely used soft ionization methods for analysis of biopolymers, matrix-assisted laser desorption/ionization (MALDI) mass spectrometry (MS) has the advantages of high sensitivity, no fragmentation of analytes, speed and ease of analysis [11,12,13,14]. However, direct MALDI- MS analysis of low-abundance biomolecules in biological origin, such as disease-associated biomarkers in serum, is still very difficult [15,16]. Thus, one or more steps to enrich the sample prior to analysis are required. However, contaminants such as buffers, salts and detergents in the solutions must be removed before the MS analysis in order to achieve the best mass spectroscopic quality. In an attempt to find the best way to solve these problems, many methods based on non-specific or specific affinity interactions have been reported for sample purification and enrichment prior to MS analysis. Many synthetic polymeric materials and nanoparticles have been applied with different pretreatment protocols [16,17]. Among them, the method based on ND is one of the most attractive, due to its advantages of sensitivity, performance and speed. Moreover, further development of surface modifications provide an extremely wide possibility for high binding selectivity for different target biomolecules, which has led to wide applications and quick development of the method in the past five years. Alternatively, NDs have been also applied into another important soft ionization method of electrospray ionization (ESI) [18] through an effective elution process [19]. Most examples in this review will show the application of NDs in the MALDI-MS, but some applications based on ESI-MS will be discussed too.

## 2. Methods

The general protocol of applying NDs in MALDI-MS analysis includes four steps [20,21]. First, an aliquot of sample solution (5−1000 µL) is mixed with the diamond suspension (1–5 µL at a concentration of 1 µg/µL) in a centrifuge tube for 2 min. Second, the NDs are separated by centrifugation at 12,000 rpm for 5 min and then the supernatant is removed. Third, the diamonds are further washed by water or other solutions in order to remove salts or detergents. Finally, an aliquot of matrix solution (1–10 µL) is mixed with the precipitate, and then the mixture is dropped on the probe for substantial MALDI-MS analysis. This is an easy and fast method, since no elution of analyte molecules from diamonds is needed before the MALDI-MS analysis. The method has also been developed to be an effective solid-phase extraction and elution platform for systematic proteome analysis, which is called as SPEED [19]. The platform facilitates purification and concentration of intact proteins and their enzymatic digests not only for MALDI-MS analysis, but also for sodium dodecyl sulfate (SDS)-polyacrylamide gel electrophoresis (PAGE) and ESI-MS analysis.

**Figure 1:**
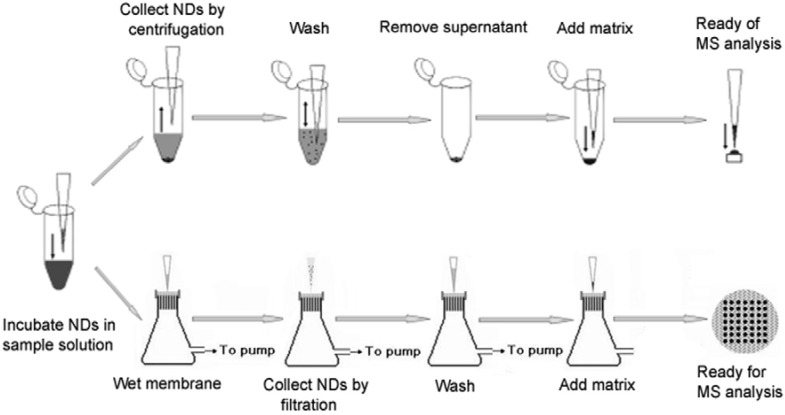
Processes of sample preparation with NDs based on centrifugation [20,21] (upper) and filtration [17] (lower) to enrich biopolymers for MALDI-MS analysis.

A new separation method based on filtration instead of centrifugation has also been developed [17]. In this method, a piece of pre-patterned paraffin is heat-pressed on a polyvinylidenedifluoride (PVDF) membrane to make a filter. The NDs are filtered out from the mixture by the PVDF membrane, followed by a washing step with water. Then the matrix solution is directly dropped on the sample spot. After drying, the whole membrane is taped on a normal MALDI probe by a double-sided adhesive tape for MS analysis. The function of the coated surface of pre-patterned paraffin is to prevent the extensive pervasion of the sample on the membrane. And hopefully this procedure is easier to automate and thus more suitable for high-throughput applications in proteomics research. Both processes are showed in Figure 1.

## 3. Applications of Carboxylated/Oxidized NDs

Carboxylated/oxidized NDs are usually prepared according to the procedures of Ushizawa *et al.* [22]. After purification by a mixture of concentrated H_2_SO_4_ and HNO_3_ at room temperature, NDs are subsequently put in 0.1 M NaOH aqueous solution for 2h and 0.1 M HCl aqueous solution for 2 h, both at 90 °C. Then they are washed, separated and then made as stock suspensions with deionized water before use as a solid phase extraction (SPE) material for MALDI-MS analysis [20,23].

### 3.1. Analysis of Peptides

**Figure 2:**
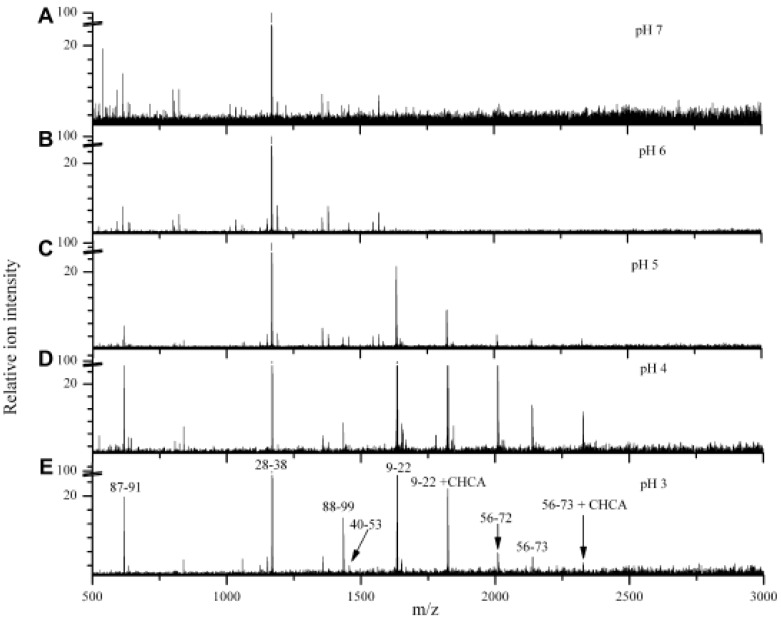
Atmospheric pressure MALDI mass spectra of cytochrome c trypsin digest on ND extraction from different pH-buffered solutions: pH 7 (A), pH 6 (B), pH 5 (C), pH 4 (D), and pH 3 (E). Reproduced from reference [24] with permission from Elsevier.

Though the ND particles can adsorb peptides quickly, the amounts of the peptides adsorbed is very sensitive to solution pH values [19,24]. The pH dependence of adsorption of different peptides is found to be relative to their pI values. Figure 2 demonstrates the varying peptide absorption behavior from cytochrome c digest solution at different pH values. The peptides with pI values greater than 9 (for example, fragment 28–38 with a theoretical pI value of 9.44) were effectively concentrated by the NDs when the solution pH was 7 or even down to 3. In contrast, the peptide 40–53, with a theoretical pI value of 5.5, can be observed only when the solution pH is adjusted to less than 5. The rule is that peptide can only be adsorbed on the ND surface when the solution is more acidic (by 1 or 2 pH units) than the pI value of the peptide. These results can be explained by the fact that the surface of carboxylated ND is found to be negative charged at solutions with pH ≥ 3, which was confirmed by ξ-potential analysis [25].

### 3.2. Analysis of Proteins

**Figure 3:**
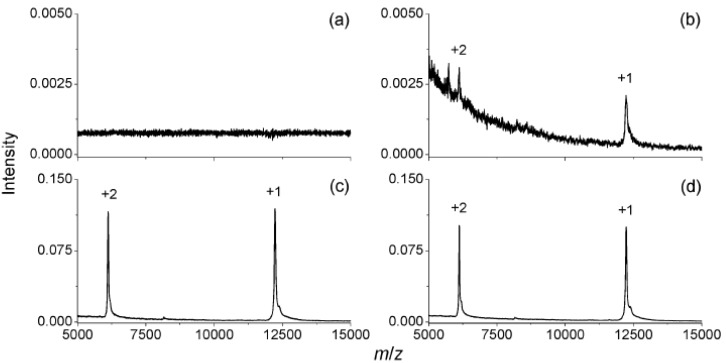
MALDI mass spectra of 100 nM bovine cytochrome c in 200 mM phosphate buffers at pH 6.5, obtained without any pretreatment (a) and with the NDs pretreatment (b). The mass spectra shown in (c) and (d) were obtained with the NDs pretreated, but before submission for MALDI mass analysis, the protein-diamond mixtures were washed once (c) and four times (d) with deionized water. Reproduced from reference [20] with permission from the American Chemical Society.

The carboxylated/oxidized NDs exhibit a remarkably high affinity for proteins, which comes from ionic interaction, hydrogen bonding, hydrophobic and van der Waals interaction [20,23,26]. The protein-surface interaction is so strong that the adsorption isotherms of proteins show a very sharp increase and saturate quickly. Unlike peptides, proteins can be adsorbed by NDs from different solutions at various pH values. However, the amount of the protein adsorbed varies with solution pH values very much, but is maximal at a pH value equal to the pI value of the corresponding protein [20]. Kong *et al.* [20] first used ND as a material to quickly enrich dilute proteins in solution before MALDI-MS analysis. They noticed that the lowest concentrations for detection can be 0.2 nM for bovine cytochrome c and horse myoglobin with the pretreatment of carboxylated/oxidized NDs (for 500 µL sample solutions with pH ~5), while these are both 100 nM (for 0.5 µL sample solution) with the conventional MALDI-MS method. In addition, it has been found that the diamonds can catch proteins from contaminated solutions readily. Figure 3 shows one example: By normal MALDI-MS analysis, no signal can be found for the solution of 100 nM cytochrome c in 200 nM phosphate buffer. However, the signals can be clearly identified when 10 µg of NDs was added to the solution and separated by centrifugation. With an additional very simple step of washing with deionized water before mixing with the matrix, the signals were further greatly enhanced. Even after rinsing with water four times, the signals remained, due to the high affinity of the diamond surface for the proteins. A further systematic study of compatibility of buffer compositions with protein adsorption to NDs was later studied by Chen *et al.* [19]. Very impressively, the affinity was not adversely affected by some severely contaminated solutions like 9M urea, 50% glycerol, or others.

### 3.3. Analysis of Body Fluids

**Figure 4:**
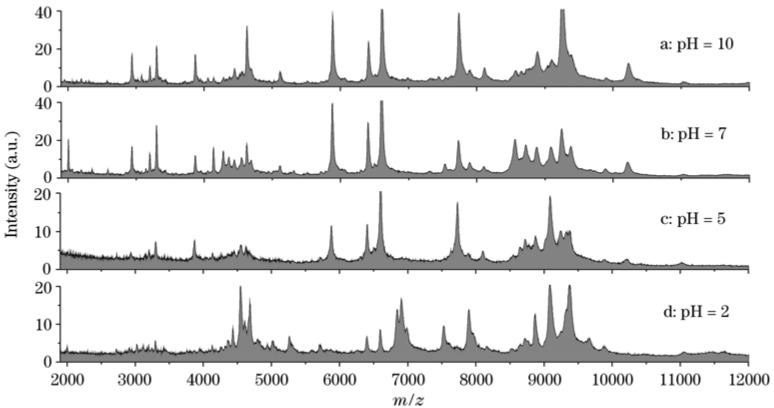
MALDI mass spectra of human serum after 100-fold dilution in phosphate buffers with different pH values and then pretreated with NDs. Reproduced from reference [21] with permission.

The MS analysis of human body fluids by MALDI or SELDI [27] has been demonstrated to be a powerful technique in searching for biomarkers that are relevant to different physiological disease states [28]. The potential application of NDs to analyze body fluids including blood serum and urine for clinical proteomics research has also been studied [19,20,21,29]. For example, direct analysis of human blood serum with regular MADLI-MS usually produces a mass spectrum with very few stable signals. With the help of NDs, it has been found that more than 80 peaks can be clearly identified in the mass range of 2000−10,000, even for just 10 μL of sample. Compared to the widely used ZipTip method [30], the acquired mass spectrum is five-fold higher in overall peak intensity and is noticeably richer in spectral features over the entire mass range. Considering the pH dependency of adsorption for peptides and proteins are both relative to their pIs, NDs hold great promise for capturing peptides and proteins selectively in mixed solutions by adjusting the pH values of the solutions [20,21]. This characteristic is valuable for the detection of low-concentration peptides and proteins, such as potential biomarkers, in body fluid analysis. By selecting suitable pH values to those ions, signals of some abundant but uninteresting ions with unmatched pI values can be greatly suppressed. Kong [21] has analyzed the same serum sample but diluted into solutions with different pH values of 2, 5, 7, and 10. It was found that the mass spectra obtained under extreme pH values were very different to those achieved at neutral surroundings (Figure 4). By selecting different matrices in MALDI-MS analysis, the potential of this method looks more attractive [21].

Urine, which is a readily obtainable biological sample, is also a very valuable example for direct MALDI-MS analysis, since the pattern of urinary protein is very useful to identify the cause of diseases that affect the function of kidneys and induce excessive losses of proteins in the urine [19,21]. However, the existence of urea and other salts makes direct MS analysis unpractical. ND can play a very important role in the urinary proteomics due to its high affinity to catch proteins and peptides from highly dilute and contaminated solutions. It can enrich proteins and peptides from very large volumes that can be separated and washed readily, which makes the whole process faster and more effective than other existing methods [19]. Results have shown that more than 70 peaks can be clearly identified in the m/z range of 2000−25,000 by direct MALDI-MS analysis of 4 μg NDs in 100 μL urine [21].

### 3.4. Other Applications

**Figure 5:**
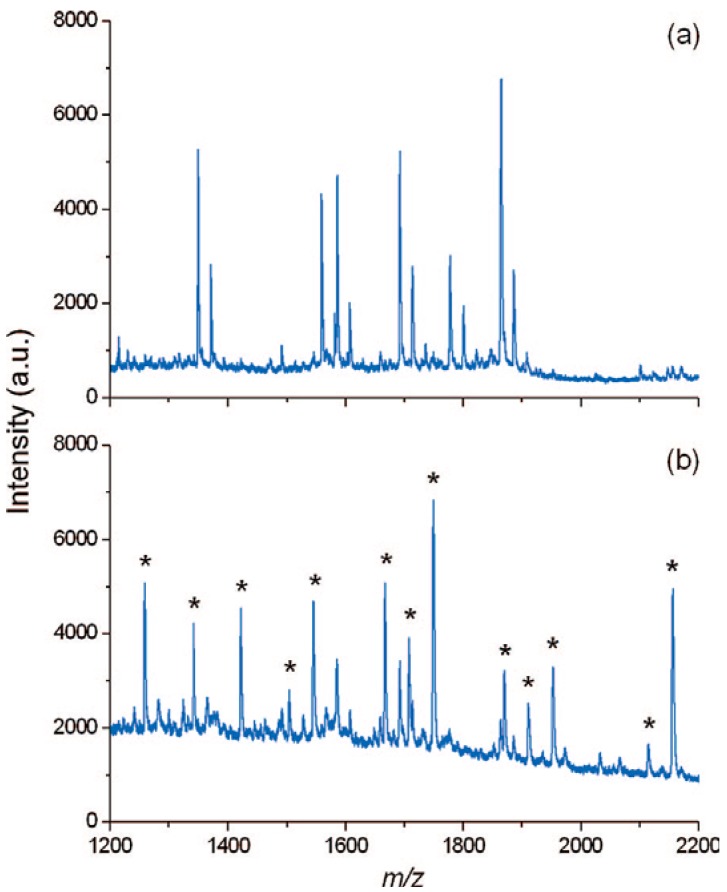
MALDI mass spectra of peptide-glycan mixtures resulting from enzymatic digestion and deglycosylation of ovalbumin (a) before and (b) after peptide depletion with carboxylated/oxidized NDs. Asterisks denote peaks derived from glycan ions. Reproduced from reference [31] with permission from the American Chemical Society.

In some cases, the extensive signals of protein and peptides in MS are really something unwelcome, since they can suppress severely the signals of objective ions in the biological samples. For example, sensitivity of neutral glycans by MALDI-MS is far less than two-orders that of peptides. Thus, an effective and rapid way to remove the proteins and peptides form the digestion solutions of glycoproteins is very important for improving MALDI-MS analysis of neutral underivatized glycans released from glycoproteins. Fortunately, Tzeng *et al.* [31] have found that the carboxylated/oxidized NDs have a much lower affinity for both neutral and acidic oligosaccharides than proteins or peptides in aqueous solution. Consequently,they setup a new protocol to accelerate the purification process for MS analysis of neutral glycans with NDs. Figure 5a shows one mass spectrum of a solution containing both the glycans released enzymatically and the tryptic digests of ovalbumin, in which all peaks are contributed exclusively by nonglycosylated peptide ions. Impressively, by depletion of the peptides with the NDs, ions arising from glycans became dominant in the mass spectrum (Figure 5b). Of course, it must be noted here that the protocol is different with the aforementioned methods, since here it is the supernatant that is analyzed instead of the NDs.

## 4. Applications of Surface-Modified NDs

### 4.1. Polylysine (PL)-Coated NDs

**Figure 6:**
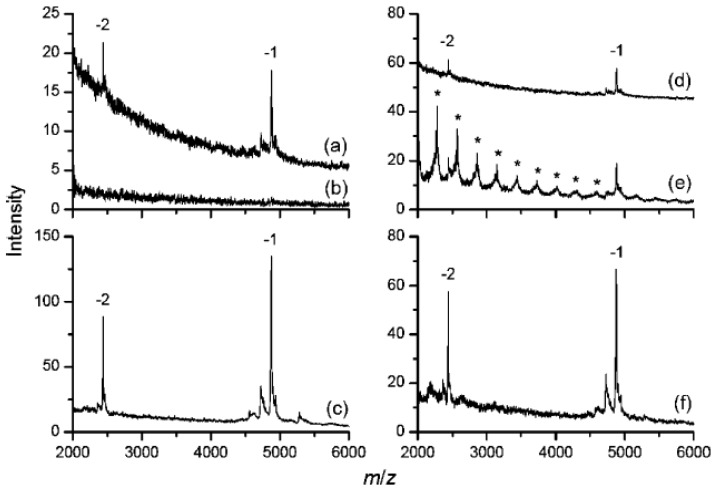
MALDI mass spectra of d(ATCGGCTAATCGGCTA) from 50 nM solutions prepared with (a) deionized water, (b) 2 M urea, and (c) 2 M urea but pretreated with PL-coated NDs prior to MS analysis. The corresponding mass spectra for sample solutions containing 0.1% SDS are shown in (d-f). Curves a and d are shifted vertically for clarity, and asterisks in (e) denote SDS cluster ions. Reproduced from reference [32] with permission from the American Chemical Society.

Huang *et al.* [23] have demonstrated that PL can be used to modify the surface of ND by non-covalent interaction. PL is highly positively charged at neutral pH and entraps CO moieties on the surface of diamond via multiple H-bondings in an extended configuration, forming a firm hydrophilic layer on the diamond surface. NDs were also coated by PL through covalent bond using 1-ethyl-3-(3-dimethylaminopropyl)-carbodiimide (EDC), and they had similar properties to those of non-covalent coated NDs [17]. Kong *et al.* [32] have found that those PL–coated NDs can serve for the detection of DNA oligonucleotides by MALDI-MS. The maximal amount of DNA molecules adsorbed to PL-coated diamonds has been found to be a function of solution pH, decreasing nearly monotonically with the increasing solution pH from 3 to 9. Since PL is protonated up to pH 10.5 and DNA oligonucleotides contain only negatively charged (phosphate) groups, such a monotonic decrease is a reflection that electrostatic forces dominate the adsorption process. Thus, in order to get a better enrichment, the pH value of the DNA solution is always adjusted to 3. Similar to carboxylated/oxidized NDs, they can be used to enrich DNA from highly contaminated solutions including slats, urea, SDS and other detergents. And the interfering signals can be effectively removed after washing with deionized water (Figure 6). Another outstanding feature of PL-coated NDs is that it can concentrate oligonucleotides selectively and preferably in the presence of an excessive amount of proteins in solution by controlling the amounts of NDs used in the process [32], although itself also can be used to enrich proteins from dilute and contaminated solutions by salts and other detergent, just like the carboxylated NDs [17].

### 4.2. Polyarginine (PA)-Coated NDs

**Figure 7:**
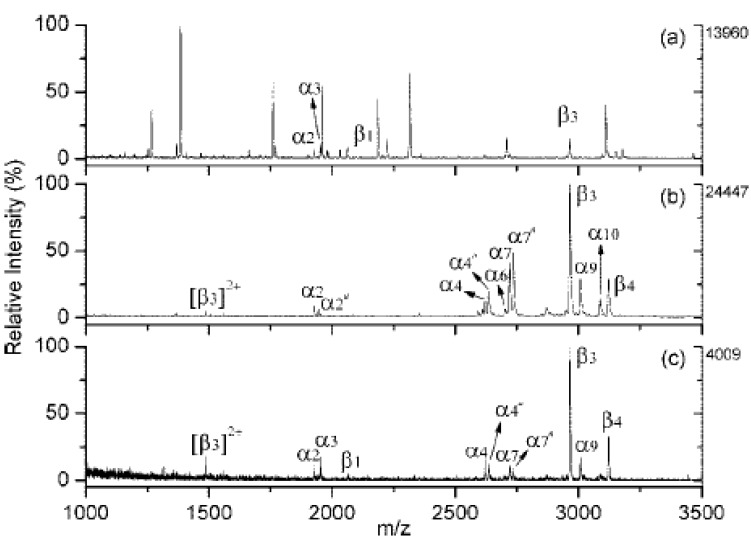
(a) Direct MALDI mass spectrum of the tryptic digest of nonfat milk with an estimated casein concentration of 2.5 × 10^-6^ M (1 μL). (b, c) MALDI mass spectra of the same sample obtained using PA-coated NDs to extract and enrich phosphopeptides from the 100 μL tryptic digest product after (b) 100-fold and (c) 1000-fold dilution. The phosphopeptides without oxidation are denoted by Arabic numerals, while the phosphopeptides containing oxidized methionine residues are marked with # in spectra b and c. Reproduced from reference [33] with permission from the American Chemical Society.

PA-coated NDs can be prepared via an EDC-mediated coupling reaction following the similar procedures used for covalent linking of PL [33]. The amino acid arginine residues are typical recognition sites in protein–protein interactions involving phosphate or sulfate moieties, and their high affinity for phosphate groups are able to generate stable complexes having “covalent-like” stability. Accordingly, Wu *et al.* [33] have proved that PA-coated NDs can be a very useful tool for isolating multiphosphorylated peptides, leading to improved detection sensitivity in the complex solutions. Figure 7 shows an example of extracting and enriching phosphopeptides from the tryptic digests of nonfat milk, which contains abundant phosphoproteins. A direct MALDI mass spectrum of the tryptic digest of this nonfat milk shows that only four peaks from more than 40 peaks are derived from phosphopetide ions (Figure 7a). However, with the help of PA-coated NDs, 12 or 11 peaks belonging to the phosphopeptide residues can be clearly identified even after dilution by 100 or 1000 times (Figure 7b, and 7c). Most attractively, the selectivity for the phosphopeptide residues is so good that only a very few weak peaks of nonphosphorylated peptides were observed in the mass spectra after the extraction and enrichment.

### 4.3. Modifications by Aminophenylboronic Acid (APBA)

**Figure 8:**
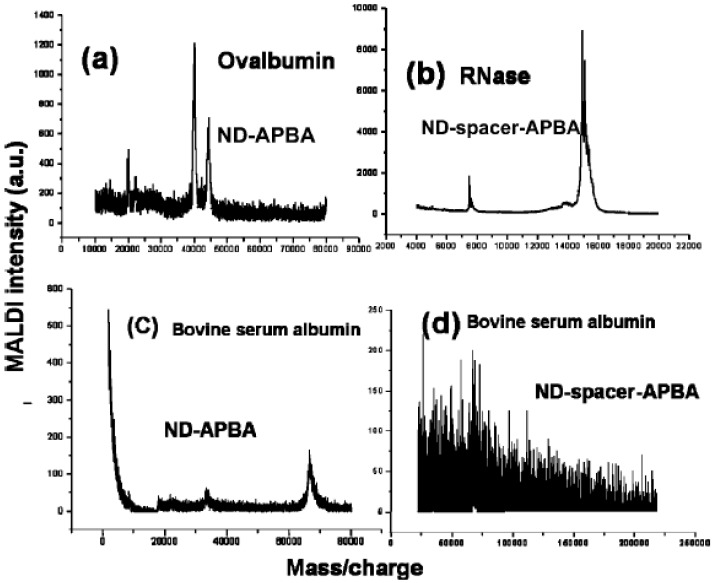
MALDI mass spectra obtained with functionalized NDs. Panels a and b show positive detection for ovalbumin and RNase, both glycoproteins, measured on ND-APBA and ND-spacer-APBA, respectively. The difference in selectivity is shown in panels c and d for the nonglycoprotein bovine serum albumin (BSA): panel c, taken on ND-APBA, shows signal for BSA, whereas panel d shows the absence of signal for BSA, attesting to the selectivity of ND-spacer-APBA for glycoproteins. Reproduced from reference [34] with permission from the American Chemical Society.

Yeap *et al.* [34] demonstrated that the functionalization of ND with aminophenylboronic acid (APBA) can selectively capture glycoproteins form unfractionated protein mixtures. Two kinds of modification have been fulfilled; in one (ND-APBA) is formed by direct immobilization of APBA on the carboxylated/oxidized NDs, and in the second (ND-spacer-APBA) is formed by immobilization of APBA after modification with a spacer chain extension on the NDs. It has been found that the nonselective binding of proteins was still obtained for ND-APBA, while ND-spacer-APBA had a better effect to enrich glycoproteins selectively, due to the role of the alkyl spacer chain to form an exclusion shell, which suppresses nonspecific binding and reduces steric hindrance among the bound glycoproteins (Figure 8).

## 5. NDs as Platforms in MS-Based Study

### 5.1. Digestion on the Surfaces of NDs

**Figure 9:**
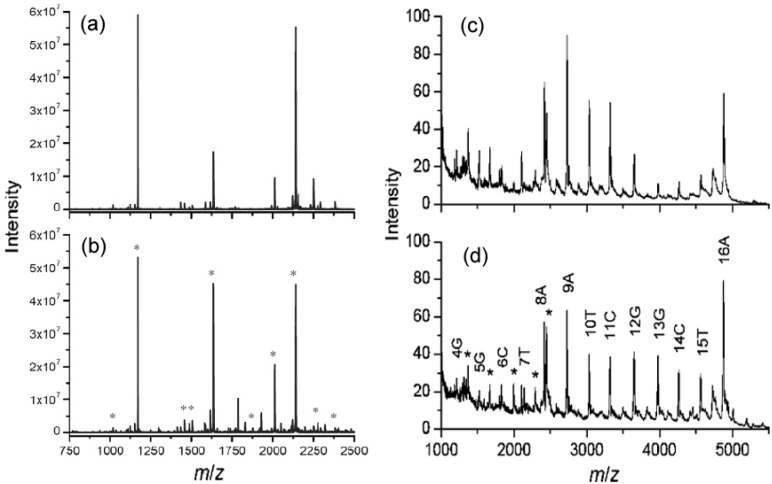
Left: MALDI mass spectra of bovine cytochrome c digested with trypsin (a) on diamond and (b) in solution. Asterisks denote the peptide masses matched to theoretical trypsinized cytochrome c fragments. Right: MALDI mass spectra of enzymatic d(ATCGGCTAATCGGCTA) digests. (c), the digestion was conducted in solution and analyzed with preconcentration using PL-coated diamonds. (d), the digestion was conducted on particle directly. Asterisks denote doubly charged DNA fragment ions. Reproduced from references [19] and [32] with permission from the American Chemical Society.

Chen *et al.* [19] found that proteolytic digestion can be conducted for proteins bound to carboxylated/oxidized NDs. The peptide mass spectra of cytochrome c digested with trypsin on NDs are very similar to those of the same protein digested in solution in terms of sequence coverage (Figure 9a and b). Kong *et al.* [32] also found that the enzymatic digestion of DNA can be conducted directly on the PL-coated NDs after the adsorption. They successfully used the acting exonuclease of snake venom phosphodiesterase (SVP) to digest the 16-mer DNA caught by the PL-coated diamonds (Figure 9c and d). Thus, the NDs or the modified NDs can be used as platforms to perform digestion on their surfaces after the concentration, which can be developed to be a standard step of “bottom-up” MS approach to identify protein or DNA by mass mapping and characterize modifications.

### 5.2. SPEED Method

**Figure 10:**
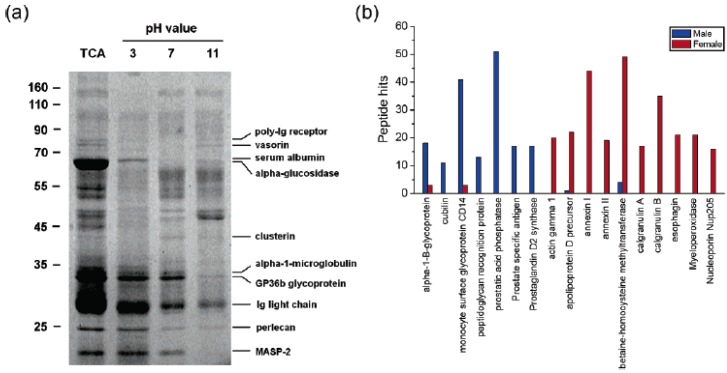
(a) SDS-PAGE analysis of urinary proteins extracted with NDs at three different solution pHs. Results from trichloroacetic acid (TCA) precipitation for the same sample are shown in the left-most lane for comparison. (b) Sex-dependent protein histogram showing peptide hits of urinary proteins identified in the urine samples from 10 healthy male (blue) and 10 healthy female (red) adults. Reproduced from reference [19] with permission from the American Chemical Society.

The method of SPEED was developed by Chen *et al.* [19], in which NDs were applied as an effective solid-phase extraction and elution platform for systematic proteome analysis by both direct SDS-PAGE and LC-ESI-MS. In the example of the analysis of urinary proteins with the SPEED platform, 125 proteins from 10 female and 10 male urine samples were identified according to 60 independent ESI-MS/MS runs. The relative abundance of the diamond-bound proteins was compared by counting the number of identified peptides. Very interestingly, the authors found that some components appear more frequently in the male than the female urine or *vice* *versa,* and thus can be identified as “sex preferential” (Figure 10). For example, calgranulin B can be only identified in female urine, which is consistent with previous findings by other methods. This appropriate identification of the sex-related proteins suggests that in combination with LC-ESI-MS/MS and SDS-PAGE , the application of SPEED platform is a very promising approach for rapid screening of biomarkers from human body fluids.

### 5.3. Non-Covalent Interaction Study

**Figure 11:**
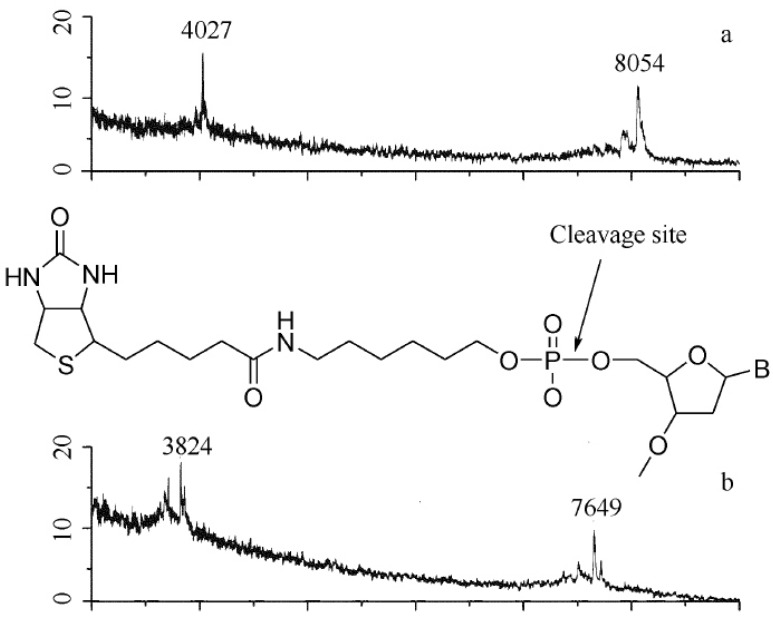
MALDI mass spectra (negative ion mode) of biotinylated T7 primer (mass 8055 Da) by (a) direct analysis of 1 μL of 0.4 μmol/L solution; (b) analysis after pretreatment by streptavidin-bound NDs. The intersection in (b) shows the cleavage site of the biotinylated DNA. Reproduced from reference [35] with permission.

It has also been noticed that NDs can be used as a platform for non-covalent study by MALDI-MS [35]. With the help of NDs, a different approach of “Bait and Fish” has been suggested to study non-covalent interactions by MALDI-MS. In this method, the “fish analyte” is separated from the solution by “bait-bound” NDs and further analyzed by MALDI-MS directly. Kong [35] used this method to show a non-specific interaction between cytochrome c and a DNA molecule of d(ATCGGCTAATCGGCTA) by direct analysis of the NDs. Since the NDs have low affinity for small molecules and high affinity to most proteins, it may be used as a primary step for high throughput drug screening for small molecules by MALDI-MS. However, strong non-covalent interaction between the “Bait and Fish” can also cause signal loss of desired ions or formation of non-expected fragmental signals, which makes the judgments difficult. A good way to solve the problem is to get one more mass spectrum of the supernatant after the pretreatment of diamonds to be compared with the original solution. Figure 11 shows the strong interaction between streptavidin and a biotinylated primer DNA by this method [35]. Though the carboxylated/oxidized NDs show no absorption for the molecule of biotinylated T7 primer in the medium solutions, streptavidin-covered NDs do, which can be verified by the MS analysis of the supernatant after the pretreatment. However, the direct MALDI–MS analysis of the diamonds showed a peak at 7649, instead of 8054, corresponding to the mass of biotinylated T7 primer ions. This can be explained by the cleavage of the OP bond linking the biotin and the DNA (with a mass loss of 405) during the process of MALDI, reflecting the strong interaction between the biotin and the streptavidin adsorbed on the surface of ND.

## 6. Other MS Study

**Figure 12:**
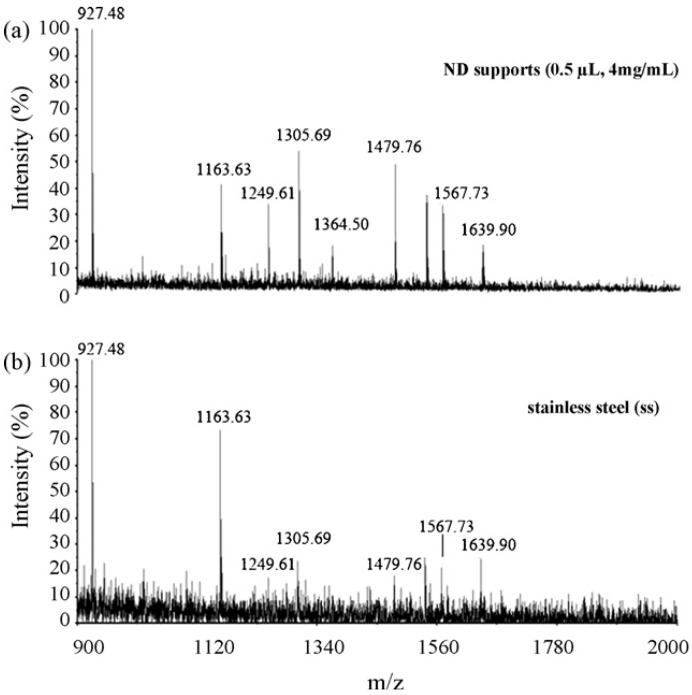
MALDI mass spectra obtained from 0.5µL of 30 nM BSA tryptic peptide. The sample was prepared on the ND support (0.5µL, 4 mg/mL) (a) and on stainless steel (SS) (b), respectively. Reproduced from reference [37] with permission from Elsevier.

Even without any modification, the detonation ND particles have also successfully been employed as an absorbent for pretreatment of peptides from diluted and contaminated solutions in a wide pH range [36]. Or they can be used as an effective and useful MALDI-MS support for protein identification [37,38]. In comparison to stainless steel, matrix crystallized more homogeneously on the ND support and thus better reproducibility of MS spectra was achieved. It has higher salt tolerance in MALDI-MS analysis and increased detection sensitivity with the reduced spot size (Figure 12). ND has been also suggested to be used as a matrix itself [39,40]. For example, Belchova *et al.* [39] has found that the suspension of fine powdered synthetic diamonds in acetonitrile can be used as an effective MALDI matrix which allows very sensitive detection of deoxynivalenol (DON). However, it should be mentioned here that if the laser energy used in MALDI-MS is too high, the singly charged carbon clusters of C_n_^+^ from ND itself can also be observed in the mass spectra [41].

## 7. What is the Next?

It is anticipated that further application of NDs in biological mass spectroscopy can be fulfilled by designed surfaces according to the specific requirements of the desired applications. Apart from carboxyl, a variety of functional groups including lactone, ketone, hydroxyl and alkyl have been present on the surfaces [42,43,44,45,46]. And by organic functionalization of the surface hydroxyl groups, Zheng *et al.* [46] have modified the surface of ND with several functional groups including halides, amines, cyanide, azide and thiols. Grafting biologically active moieties, such as ligands, peptides, antibodies, enzymes and DNA oligonucleotides have also been applied to the surfaces of NDs [47,48,49,50,51], making the enrichment and detection of a variety of molecules including biomarkers performable. All these, as well as other new modifications, can greatly extend the potential applications of NDs in biological MS analysis in the near future. On the other hand, the new biomolecular MS technology such as new ionization method of desorption electrospray ionization [52] and MS image [53] provide more application possibilities for NDs.

## 8. Conclusions

ND can be readily used as a SPE material for enriching peptides and proteins for direct MALDI-MS analysis or ESI-MS analysis after elution. The high affinity of carboxylated/oxidized NDs to peptides and proteins makes the direct MALDI-MS analysis of complex biosamples to be feasible. With different polymers coated on the surfaces, the application can be further expanded very much, as the examples of PL-coated and PA-coated NDs in the analysis of DNA and multiphosphorylated peptides. Modification with APBA after extention by a spacer chain improves its ability for selective capture of glycoprotein manifestly. And direct MS analysis of biological samples including body fluids such as serum and urine, shows its great potential in the field of proteomics. We hope that this review can encourage further applications of NDs with more designed modifications for biological MS analysis, so as to improve its sensitivity, selectivity, throughput and automation, for making full use of the advantage and potential of NDs in fields of analytical chemistry and molecular biology.
